# Neuroprotective Nanoparticles Targeting the Retina: A Polymeric Platform for Ocular Drug Delivery Applications

**DOI:** 10.3390/pharmaceutics15041096

**Published:** 2023-03-29

**Authors:** Patrizia Colucci, Martina Giannaccini, Matteo Baggiani, Breandán N. Kennedy, Luciana Dente, Vittoria Raffa, Chiara Gabellini

**Affiliations:** 1 Department of Biology, University of Pisa, 56127 Pisa, Italy; 2 Department of Biotechnology, Chemistry and Pharmacy, University of Siena, 53100 Siena, Italy; 3 UCD Conway Institute, University College Dublin, D04 V1W8 Dublin, Ireland; 4 UCD School of Biomolecular and Biomedical Science, University College Dublin, D04 V1W8 Dublin, Ireland

**Keywords:** polymeric nanoparticles, drug delivery and targeting, ocular posterior segment, oxidative stress, retinal degeneration, nerve growth factor, peanut agglutinin, zebrafish

## Abstract

Neuroprotective drug delivery to the posterior segment of the eye represents a major challenge to counteract vision loss. This work focuses on the development of a polymer-based nanocarrier, specifically designed for targeting the posterior eye. Polyacrylamide nanoparticles (ANPs) were synthesised and characterised, and their high binding efficiency was exploited to gain both ocular targeting and neuroprotective capabilities, through conjugation with peanut agglutinin (ANP:PNA) and neurotrophin nerve growth factor (ANP:PNA:NGF). The neuroprotective activity of ANP:PNA:NGF was assessed in an oxidative stress-induced retinal degeneration model using the teleost zebrafish. Upon nanoformulation, NGF improved the visual function of zebrafish larvae after the intravitreal injection of hydrogen peroxide, accompanied by a reduction in the number of apoptotic cells in the retina. Additionally, ANP:PNA:NGF counteracted the impairment of visual behaviour in zebrafish larvae exposed to cigarette smoke extract (CSE). Collectively, these data suggest that our polymeric drug delivery system represents a promising strategy for implementing targeted treatment against retinal degeneration.

## 1. Introduction

Diseases affecting the posterior segment of the eye, including glaucoma, age-related macular degeneration (AMD) and retinopathies, represent a debilitating and increasingly frequent worldwide health problem, leading to partial or complete blindness [1]. Oxidative stress plays a key role in the pathogenesis of ocular degenerative diseases. In fact, the retina is extensively exposed to light and is a highly metabolic tissue requiring enormous oxygen consumption. Consequently, this tissue is strongly affected by oxidative stress [2]. Moreover, in vitro studies showed that exposure to cigarette smoke extract (CSE), a mixture of genotoxic and carcinogenic compounds, induced oxidative stress in human retinal cells and injured primary retinal ganglion cells in rats [3,4]. Systemic treatment with CSE alters the retinal structures as well as the visual behaviour of zebrafish larvae [5], thus furthering the contribution of smoking in the pathogenesis of degenerative diseases.

Neurotrophins are extensively involved in the molecular response against oxidative damage [6,7,8]. Nerve growth factor (NGF), the most well-characterised member of the family, as well as its receptors are widely expressed in the visual system, thus suggesting a crucial role in promoting retinal development and cell survival [9,10]. Several experimental studies demonstrated that NGF exerts neuroprotective and regenerative effects on multiple retinal cell populations [11,12,13,14] through the modulation of several molecular pathways [15,16]. However, the clinical applicability of neurotrophins such as NGF is limited by rapid degradation and clearance in vivo [17]. Moreover, the delivery of drugs to the posterior segment of the eye is a challenging goal due to the anatomical and physiological barriers that protect this organ [18]. Nanoscale materials represent a minimally invasive method in ophthalmology for overcoming such limitations, offering a wide range of physicochemical characteristics to increase the half-life and solubility of drugs, thus improving their pharmacokinetic and pharmacodynamic profiles [19,20]. One of the most appealing characteristics of a nanodevice for the ocular posterior segment is the capability to accumulate in a specific retinal layer and to be retained for a sustained drug release over time, hence reducing the risks associated with multiple injections and side effects caused by a bolus dosage of drugs. Indeed, the number of patents for nanotechnology-based formulations used for ocular drug delivery is increasing and includes a wide range of materials with different properties [21]. However, the development of patient-friendly drug delivery systems for controlled release of therapeutics at the posterior segment of the eye is still a major challenge [22]. 

The teleost zebrafish (*Danio rerio*) represents an appealing model organism to study human degenerative diseases in a complex biological system and to validate new therapeutic strategies for the posterior segment of the eye. Interestingly, the zebrafish eye shares equivalent anatomical and cell-layered retinal structures with humans, and visual function is well established after only 3 days post-fertilisation (dpf), thus allowing short-term studies [23,24,25]. Giannaccini and coauthors demonstrated that magnetic nanoparticles (MNPs) are able to localise at the ocular posterior segment after intravitreal injection in *Danio rerio* (zebrafish) larvae [26,27,28]. In a retinal degeneration model previously developed by our group, protection against oxidative stress by NGF as well as by brain-derived neurotrophic factor (BDNF) is notably improved when they are bound to MNPs compared to the free neurotrophins, which have short half-lives and weak efficacy after in vivo administration. Thus, MNPs not only are good carriers to reach the retinal layers, but they also protect molecules from fast degradation [28]. 

Although MNPs are biocompatible and already approved for use in humans, they pose some concerns because of their inorganic nature [29,30]. Organic-based polymeric nanoparticles are the most used nanosystems to overcome these limitations. They can be developed from natural and synthetic biocompatible polymers and exploited in ocular drug and gene delivery [31,32,33]. Among them, polymeric acrylic nanoparticles possess exclusive properties that make them suitable for drug delivery applications [34,35]. The high stability and biocompatibility of polyacrylic derivatives have been widely studied, and the presence of numerous functional groups perfectly addresses the need to load drugs and enable sustained release, further enhanced by their unique properties of being responsive to external and internal stimuli [36,37].

Here, we describe a polymeric nanocarrier based on acrylic monomers, specifically designed for targeting the ocular posterior segment thanks to functionalisation with peanut agglutinin (PNA). In particular, polyacrylamide nanoparticles (ANPs) were synthesised and characterised, and their high binding efficiency was exploited for implementing a polymer-based, ocular-targeting, neuroprotective drug delivery system. The biological activity of the ANP:PNA-conjugated NGF as well as its neuroprotective efficacy have been validated in vitro. To deeply investigate the protective effect of our nanoformulation as a therapeutical strategy for the ocular posterior segment, we performed in vivo studies in zebrafish. Using the previously established zebrafish model of visual impairment induced by oxidative stress [28], we demonstrated by both behavioural and functional assessments in zebrafish larvae that only the ANP:PNA-conjugated NGF, but not the free neurotrophin, is able to protect the retina from the oxidative damage induced by the intravitreal injection of hydrogen peroxide. We additionally assessed our NGF-coated polymeric nanocarrier as a neuroprotective treatment against the visual impairment of zebrafish larvae caused by the pharmacological exposure to CSE, using a model recently described [5]. Collectively, these data suggest that our polymeric drug delivery system represents a promising strategy for implementing treatment against retinal degeneration.

## 2. Materials and Methods

### 2.1. Chemicals

*N*-Isopropylacrylamide (NIPAM; #731129), acrylamide (AAm; #A9099), allylamine hydrochloride (AH; #735132), *N*,*N*′-Methylenebisacrylamide (BIS; #M1533), sodium dodecyl sulfate (SDS; #436143), ammonium persulfate (APS; #A3678), N,N,N′,N′-Tetramethyl ethylenediamine (TEMED; #T9281), FITC-conjugated PNA from *Arachis hypogaea* (PNA; #L7381), poly-L-lysine (#P4707), DMEM F-12 Ham (#D6421), hydrogen peroxide (H_2_O_2_; #H1009), thiazolyl blue tetrazolium bromide (#M5655) and dimethyl sulfoxide (DMSO; #276855) were purchased from Merck (Darmstadt, Germany). Fluorescent dyes Alexa Fluor 488 (#A20000), Alexa Fluor 594 (#A20004), Hoechst 33342 (#H3570) and secondary antibody (#A21244) were purchased from Invitrogen (Carlsbad, CA, USA). Native mouse NGF 2.5 S protein (NGF; #N-100) was acquired from Alomone labs (Jerusalem, Israel). Dulbecco’s Modified Eagle’s Medium (DMEM; #21969), horse serum (HS; #16050), fetal bovine serum (FBS; #10270), penicillin/streptomycin (Pen Strep; #15140), GlutaMAX™ (#35050) and L-Glutamine (#25030) were purchased from Gibco (Thermo Fisher Scientific, Waltham, MA, USA). OCT embedding compound for cryostat (#05-9801) was acquired from Bio-Optica (Milano, Italy). Cigarette smoke extract (CSE) was purchased from Murty Pharmaceuticals (Lexington, KY, USA). Cleaved caspase-3 primary antibody (#9661) was acquired from Cell Signaling Technology (Danvers, MA, USA).

### 2.2. Nanoparticle Synthesis

#### 2.2.1. Synthesis of Fluorescent Polyacrylamide Nanoparticles (ANPs)

Fluorescent polyacrylamide nanoparticles were synthesised by optimising the radical polymerisation protocol developed by Rahimi and colleagues [38]. A total of 222 mg of NIPAM, 28.6 mg of AAm, 76 mg of AH and 262 μL of BIS were dissolved in 10 mL of deionised water previously purged with argon at room temperature and under stirring. Next, 11.5 μL of 10% SDS solution was added, and the solution was purged with argon for 30 min. Then, 1 mg of Alexa Fluor 488 or Alexa Fluor 594 dyes was dissolved to stop the argon flow, and 15.6 mg of APS and 20 μL of TEMED were added. Finally, deionised water was added to the final volume of 20 mL, and the reaction was carried out at room temperature for 3 h under continuous stirring in darkness. Dialysis was performed by transferring the sample into a 10 kDa cut-off membrane kept in deionised water under stirring and replaced with new water 4 times per hour. Finally, the sample was concentrated in a 30 kDa Vivaspin^®^ tube (Sartorius, Goettingen, Germany) by centrifuging at 3000× *g* until 1 mL of nanoparticle suspension was obtained, and then kept at 4 °C.

#### 2.2.2. Functionalisation of Nanoparticles with Peanut Agglutinin (ANP:PNA) and Nerve Growth Factor (ANP:PNA:NGF)

ANPs coated with FITC-conjugated PNA (ANP:PNA) were obtained through a noncovalent functionalisation of the particles by incubating a 1:1 ratio of ANPs and PNA at 4 °C for 1.5 h under stirring. The sample was concentrated using a 100 kDa Vivaspin^®^ tube (Sartorius), washed with deionised water and kept at −20 °C in a 20% glycerol solution, while the supernatant derived from the washing steps was stored for further analysis. The functionalisation with NGF and PNA was carried out by incubating a 1:1:1 ratio of ANPs, PNA and NGF (ANP:PNA:NGF), respectively, following the same protocol as above.

### 2.3. Nanoparticle Characterisation

The concentration of PNA conjugated to the ANPs was estimated by BCA assay (Pierce^TM^ BCA Protein Assay Kit; #23227, Thermo Scientific, Waltham, MA, USA), according to the manufacturer’s protocol. An ELISA assay (Mouse beta-NGF ELISA Kit; #RAB1119, Merck, Darmstadt, Germany) was performed to evaluate the amount of NGF bound to the ANP:PNA. Both quantifications were performed by using the supernatant derived from the washing steps of the functionalisation reactions. The concentration of PNA and NGF attached to the ANPs’ surface was extrapolated through subtraction by measuring the supernatants’ absorbance at 562 and 450 nm, respectively. The protein loading efficiency of the ANPs was calculated by using the following formula: (mass of protein in nanoformulation/total mass of protein) × 100.

The size distribution and polydispersity index of naked, single and double-functionalised ANPs were assessed by dynamic light scattering (DLS) using a Zetasizer NS (Malvern Panalytical, Malvern, UK).

### 2.4. In Vitro Studies

#### 2.4.1. Assessment of ANP:PNA:NGF Bioactivity 

Rat pheochromocytoma PC12 cells were obtained from the American Type Culture Collection (ATCC). Cells were cultured in DMEM supplemented with 10% HS, 5% FBS, 100 IU/mL penicillin, 100 μg/mL Pen Strep and 1% GlutaMAX™ in T25 flasks coated with 10 μg/mL poly-L-lysine. Cells were incubated in a saturated humidity atmosphere at 37 °C and 5% CO_2_. For the differentiation, PC12 cells were seeded at low density (2.5 × 10^4^ cells per cm^2^) and maintained in DMEM with 1% FBS and 100 ng/mL NGF or ANP:PNA:NGF for 4 days. Cells untreated or incubated with ANP:PNA were used as controls. Then, cells were washed with DPBS and fixed in 2% paraformaldehyde (PFA) at room temperature for 10 min. Images were acquired at 10× magnification with a Nikon Eclipse TE2000-U microscope, and the length of 180 neurites (randomly selected) was evaluated by using the plugin NeuronJ available on ImageJ software (version 1.53t). Independent experiments were performed, *n* = 3.

#### 2.4.2. Assessment of ANP:PNA:NGF Protective Effect

Human retinal pigment epithelial ARPE-19 cells were obtained from the American Type Culture Collection (ATCC). Cells were maintained in DMEM F-12 Ham supplemented with 10% FBS, 2% Pen Strep and 2 mM L-Glutamine in a humidified atmosphere at 37 °C and 5% CO_2_. Cells were seeded at density of 10 × 10^3^ cells per well in a 96-well plate. The next day, cells were co-incubated with 100 ng/mL of free or ANP:PNA-conjugated NGF and 300 µM of H_2_O_2_. Untreated cells were used as negative control, while the positive control group was only treated with 300 µM of H_2_O_2_. After 24 h, the treatment solution was removed, and an MTT assay was performed. Briefly, 100 µL of 0.5 mg/mL thiazolyl blue tetrazolium bromide solution was added, and cells were incubated for 2.5 h. Then, 100 μL of DMSO was added and incubated for a few minutes on a plate shaker to dissolve the water-insoluble formazan salt. Quantification was carried out using a CLARIOstar microplate reader, and the absorbance was measured at 595 nm. Independent experiments were performed, *n* = 3.

### 2.5. In Vivo Studies Using Zebrafish

#### 2.5.1. Animal Handling

Animals were handled in compliance with protocols approved by the Italian Ministry of Public Health, the local Ethical Committee of the University of Pisa and the University College Dublin Animal Research Ethics Committee (AREC), in conformity with EU legislation (Directive 2010/63/EU). Zebrafish embryos were obtained by natural mating of wild-type fish and maintained in the incubator at 28 °C in E3 media according to the ZFIN procedures. Before any injection at 3, 4, and 5 days post-fertilisation (dpf), larvae were anesthetised in 0.02% tricaine and embedded in 0.3% agarose.

#### 2.5.2. Nanoparticle Localisation Studies 

A total of 2 nL of green fluorescent Alexa Fluor 488-loaded ANPs or DPBS, as control, were intravitreally (IVT) microinjected into the left eye of 3 dpf larvae. Larvae were firstly observed under a stereomicroscope using both bright field and FITC channel to evidence the Alexa Fluor 488 fluorescence in vivo. Then, they were fixed after 4, 8 and 24 h post-injection (hpi) in a 4% PFA solution at room temperature for 10 min, embedded in OCT and sliced with a cryostat, obtaining 10 µm cross-sections. The sections were stained with Hoechst 33342 (1:1000) to distinguish the retinal layers and imaged with a Nikon Eclipse-Ti microscope at 20× magnification. The presence of green fluorescent nanoparticles at the ganglion cell layer (GCL), inner nuclear layer (INL), photoreceptors layer (PR) and retinal pigment epithelium (RPE) was qualitatively evaluated. The retinal distribution of ANPs was assessed on 15 larvae per group, *n* = 3.

To evaluate the effect of PNA conjugation on nanoparticle localisation, 2 nL of naked Alexa Fluor 594-positive ANPs or FITC-labelled ANP:PNA or DPBS, as control, were IVT microinjected into the left eye of larvae at 3 dpf. The injected eyes were in vivo imaged at 4 and 24 hpi with a Nikon SMZ18 stereomicroscope, using bright field, TRITC and FITC channels to check the fluorescence of Alexa Fluor 594 and the PNA: FITC-conjugated, respectively. Independent experiments were carried out on 15 larvae per group, *n* = 3. 

#### 2.5.3. Neuroprotection Studies

A volume of 2 nL, corresponding to 1 ng of free NGF or ANP:PNA:NGF or DPBS, was IVT microinjected into the left eye of larvae at 4 dpf. At the later stage of 5 dpf, larvae were injected in the same eye with 2 nL of 1 M H_2_O_2_ to induce oxidative damage as previously described by Giannaccini and coauthors [28]. In addition, 2 nL of DPBS was injected as negative control.

Similarly, 1 ng of free NGF or ANP:PNA:NGF or DPBS was IVT microinjected into one eye of zebrafish larvae at 4 dpf. After 16 hpi, larvae were immersed in E3 embryo medium containing 20 µg/mL cigarette smoke extract (CSE) or 0.05% DMSO as control for 24 h, following the protocol described by Gómez Sánchez and colleagues [5]. 

#### 2.5.4. Optokinetic Response (OKR) Assay

Visual function was assessed 8 h after the injection of hydrogen peroxide via optokinetic response (OKR) assay, as previously described [39]. Briefly, the larvae were embedded in 6% methylcellulose in a 3.5 mm Petri dish and then were placed in the centre of a drum with black and white stripes rotating at 11 rpm, and the ocular movements (saccades) of the left eye per minute were quantified. Each experiment was performed on at least 9 larvae per group, *n* = 4.

Optokinetic response was assessed 24 h after the exposure to CSE, according to the protocol previously published by Gómez Sánchez and collaborators [40]. Each experiment was performed on at least 8 larvae per group, *n* = 3. 

#### 2.5.5. Whole-Mount Immunostaining

At 8 h after the induction of oxidative stress, larvae were fixed in 4% PFA overnight at 4 °C and maintained in bleach solution (0.18 M KOH and 3% H_2_O_2_ in ddH_2_O) at room temperature for 40 min to remove the pigmentation of the RPE. The whole-mount immunostaining was carried out following an optimised protocol for zebrafish retinal samples from Inoue and Wittbrodt [41]. To enhance tissue permeabilisation, larvae were first heated in a 150 mM Tris-HCl pH = 9 solution at 70 °C for 15 min and then incubated with acetone at −20 °C for 20 min. Then, they were blocked (10% goat serum, 0.8% TritonX, 1% BSA and 1% DMSO in 1X PBS + 0.1% Tween) at 4 °C for 3 h and incubated with cleaved caspase-3 primary antibody (1:250) diluted in 1% goat serum, 0.8% Triton X-100 and 1% BSA in 1X PBS + 0.1% Tween at 4 °C for three days. Samples were sequentially washed, then incubated with the secondary antibody (1:500) and Hoechst 33342 (1:100) in the dark for two and half days. After washing, larvae were mounted on glass slides using Aqua/PolyMount and a #1.5 glass coverslip. All images were acquired by a Nikon A1 confocal microscope at 40× magnification with 2.5 μm interval Z-stacks. Quantification of active caspase 3 (aCasp3)-positive cells was performed using ImageJ software (version 1.53t) (Figure S1). Each experiment was performed on at least 6 larvae per group, *n* = 3.

### 2.6. Statistical Analysis

All data were reported as the mean ± standard error of the mean and analysed on GraphPad Prism 7. Neurites’ length was analysed by t-test for unpaired data followed by Kolmogorov–Smirnov’s test. Cell viability was analysed by one-way ANOVA followed by Tukey’s multiple comparisons test. OKR data were analysed by Kruskal–Wallis’s test, followed by Dunn’s test, one-way ANOVA and finally by Tukey’s test. Apoptosis data were analysed by one-way ANOVA followed by Dunnett’s test. Normal distribution of differences was tested by the D’Agostino and Pearson test, and significance levels were *p* < 0.05 (*), *p* < 0.01 (**), *p* < 0.001 (***) and *p* < 0.0001 (****).

## 3. Results

### 3.1. Nanoparticle Characterisation

To exploit polyacrylamide nanoparticles as nanocarriers for ocular drug delivery application, peanut agglutinin (PNA) and nerve growth factor (NGF) were conjugated to the nanocarrier through noncovalent functionalisation. PNA, a lectin from *Arachis hypogea*, is widely known to selectively recognise and bind cone photoreceptors [42], while NGF is a neurotrophin with established activity in counteracting retinal degeneration [11,12,13]. To estimate the loading of proteins onto the nanoparticles, BCA and ELISA assays were carried out for quantifying conjugated PNA and NGF, respectively. The PNA and NGF binding efficiency was 62% and 91%, respectively, as the loaded content of proteins was 0.62 µg of PNA and 0.91 µg of NGF per µL of particles suspension. 

**Figure 1:**
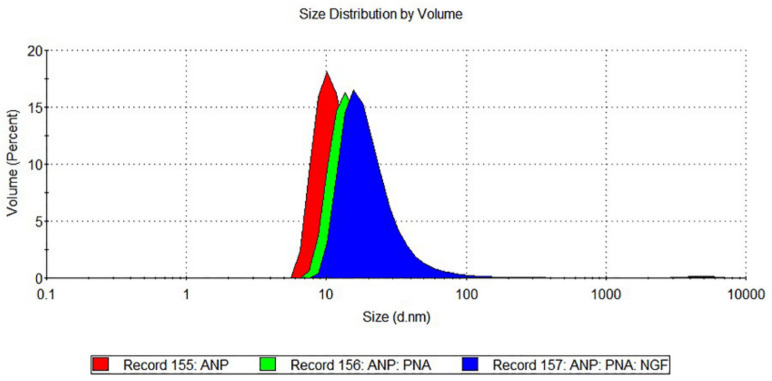
Nanoparticle characterisation through dynamic light scattering measurements. The hydrodynamic diameter increase of single- (ANP:PNA, green curve) and double- (ANP:PNA:NGF, blue curve) functionalised polyacrylamide nanoparticles resulted in a shift of size distribution compared to naked ANPs (red curve).

Size distribution and polydispersity index of the functionalised nanoparticles were also characterised by DLS measurements (Figure 1). 

ANP:PNA:NGF showed an increase in size distribution (22.81 ± 3.17 nm) compared to the PNA-conjugated ANPs (19.97 ± 2.41 nm) and naked ANPs (13.89 ± 1.74 nm), as well as an improvement in the polydispersity index that was 0.18 ± 0.03 for ANP:PNA:NGF, 0.47 ± 0.09 for ANP:PNA and 0.44 ± 0.03 for the naked ANPs (Table 1).

### 3.2. Peanut Agglutinin Is Essential for Targeting the Ocular Posterior Segment In Vivo

**Figure 2:**
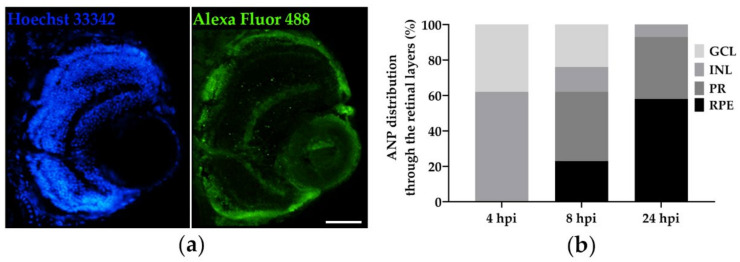
Nanoparticle distribution through retinal layers after intravitreal injection in zebrafish larvae. (**a**) Representative images of a retinal cross-section of a zebrafish larva IVT injected with Alexa Fluor 488-positive polyacrylamide nanoparticles, analysed at 4 h post-injection (hpi). Hoechst staining allows identification of the retinal layers. In FITC channel, the intense green spots clearly highlight the presence of Alexa Fluor 488-positive nanoparticles in the injected eye. (**b**) Quantitative evaluation of the distribution of Alexa Fluor 488-positive polyacrylamide nanoparticles in the retinal layers at different time points. *n* = 15 injected eyes from 3 independent experiments. GCL, ganglion cell layer; INL, inner nuclear layer; PR, photoreceptors; RPE, retinal pigment epithelium. Scale bar: 50 µm.

To study the localisation of the polymeric unbound nanocarrier and investigate its retinal distribution over time, 3 dpf larvae were IVT injected with ANPs containing the green fluorescent dye Alexa Fluor 488, then fixed at 4, 8 and 24 hpi, cryosectioned and imaged in DAPI and FITC channels (Figure 2a). By analysing cross-sections at different time points, we observed a different profile of particle distribution through the retinal layers over time. At 4 hpi, green fluorescent spots, corresponding to Alexa Fluor 488-positive ANPs, were found to localise at both the GCL and INL. At 8 hpi, they were also observed at the PR and the RPE layers, where the Alexa Fluor 488 signal was mainly present at 24 hpi and no particles were found at the GCL, thus suggesting their spontaneous migration over time (Figure 2b). Interestingly, no ANPs were found in the right eye used as uninjected control (Figure S2).

**Figure 3:**
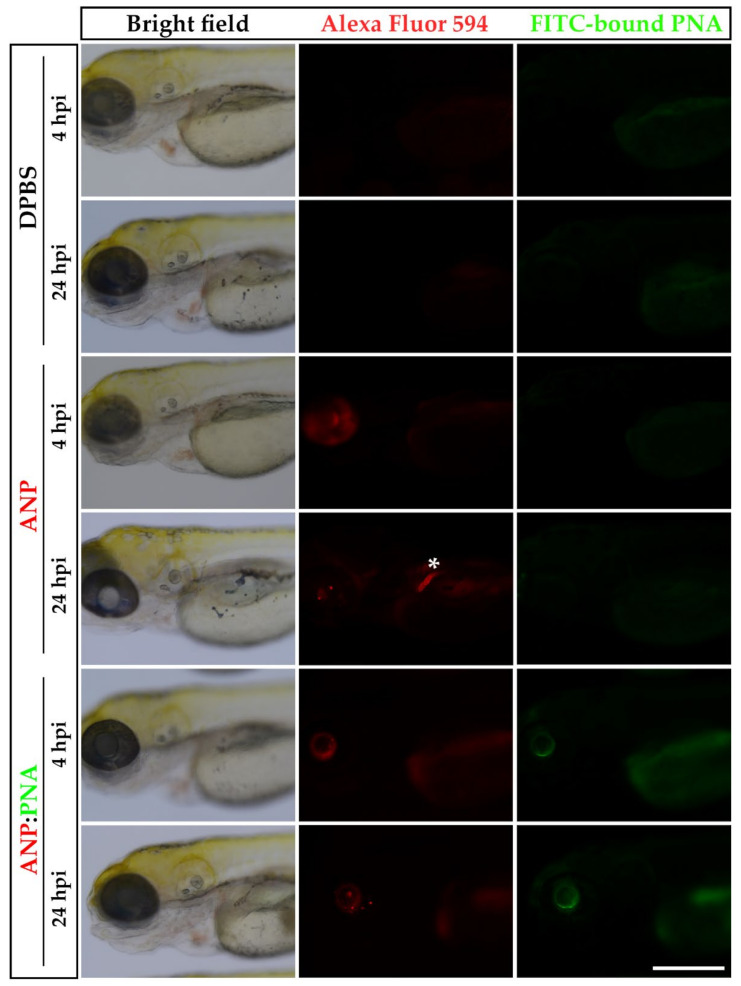
Prolonged localisation of nanoparticles at the site of injection. Representative images of 3 days post-fertilisation (dpf) larvae IVT injected with naked Alexa Fluor 594-positive nanoparticles (ANPs) or FITC-labelled PNA-conjugated polyacrylamide nanoparticles (ANP:PNA), analysed at 4 and 24 h post-injection (hpi). As control, zebrafish larvae were IVT injected with DPBS. White asterisk indicates the pronephros. *n* = 45 injected eyes from 3 independent experiments. Scale bar: 300 µm.

Nevertheless, preliminary in vivo whole-mount imaging suggested the presence of green fluorescent ANPs not only in the injected eye but also at the pronephros, where they already accumulated by 4 hpi, indicating that the nanocarrier was not stably retained in the ocular environment. To confirm that the PNA conjugation enhances the ANPs capability to target the ocular posterior segment, nanoparticles containing a red fluorescent Alexa Fluor 594 dye were functionalised with an FITC-conjugated PNA (ANP:PNA) in order to identify the distribution profile of the carrier (red fluorescent ANPs) and the cargo (green fluorescent PNA). Naked and PNA-conjugated ANPs were IVT injected in 3 dpf larvae, and the fluorescence was checked after 4 and 24 hpi (Figure 3). After 4 hpi, a strong red signal was observed in the left eye of both injected groups. However, only the larvae injected with the naked ANPs showed the presence of nanoparticles at the level of the pronephros at 24 hpi, confirming the evidence previously described and suggesting their instability in the ocular environment. On the contrary, the injected PNA-conjugated ANPs were retained in the eye until 24 hpi, as observed in 100% of injected larvae. Indeed, the stable localisation of ANP:PNA was indicated by the presence of green fluorescence of the FITC-conjugated PNA in the left eye at all the time points and its absence in any other tissue of the larvae, thus suggesting a crucial improvement of the polymeric nanoformulation as an ocular drug delivery nanocarrier. No fluorescence was found in control larvae injected with saline solution, as expected (Figure 3).

### 3.3. Nanoformulated NGF Preserves Human Retinal Cells Viability in Oxidative Conditions

**Figure 4:**
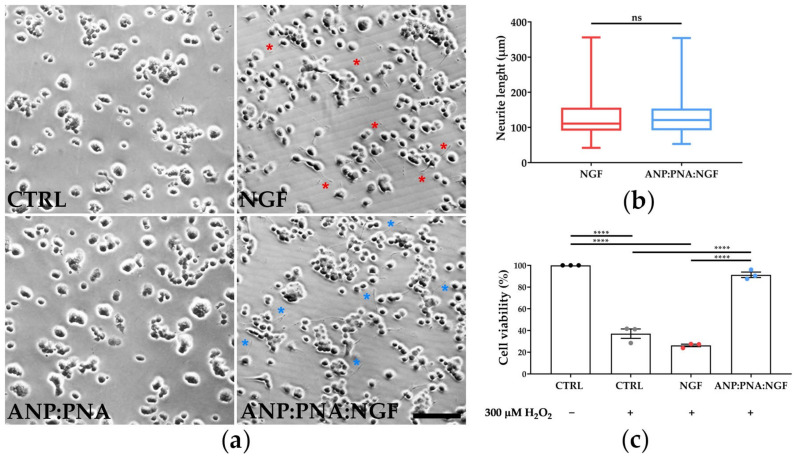
NGF exerts its biological activity upon nanoformulation. (**a**) Representative images of PC12 cells incubated for 4 days with 100 ng/mL of free NGF or with the same concentration of NGF bound to PNA-conjugated polyacrylamide nanoparticles (ANP:PNA:NGF). As control, PC12 cells were incubated with PNA-conjugated nanoparticles (ANP:PNA) or DPBS. Red and blue asterisks indicate the presence of neurites in cells exposed to NGF and ANP:PNA:NGF, respectively. Scale bar: 300 µm. (**b**) Quantitative evaluation of neurite length in PC12 cells incubated with NGF or ANP:PNA:NGF. *n* = 180 neurites from 3 biological replicates, t-test for unpaired data followed by Kolmogorov–Smirnov test. (**c**) Protective effect of NGF-conjugated polyacrylamide nanoparticles against oxidative stress. ARPE-19 cells were cotreated with 100 ng/mL of free NGF (NGF) or nanoformulated NGF (ANP:PNA:NGF) and 300 µM of H_2_O_2_ for 24 h. MTT assay was performed to evaluate cell viability. Absorbance values of all the groups were normalised to those of the untreated control sample (CTRL), which was set at 100%. *n* = 3 biological replicates, one-way ANOVA applying Tukey’s multiple comparisons test. (**b**,**c**) ns, *p* > 0.05 and ****, *p* < 0.0001.

The biological activity of the ANP:PNA-conjugated NGF was assessed on pheochromocytoma PC12 cells, since they differentiate in a neuron-like phenotype in response to the NGF treatment [43]. After 4 days of treatment, PC12 cells displayed the presence of long neurites in both groups treated with free or conjugated NGF, thus confirming the NGF bioactivity through its ability of triggering neurite formation (Figure 4a). As expected, untreated and ANP:PNA-treated control cells maintained a round-shape morphology. Additionally, neurite length was comparable between the groups exposed to free NGF (127.5 ± 4.17 µm) and the nanoformulation (129 ± 3.74 µm), as shown in Figure 4b, suggesting that the functionalisation process did not affect the biological activity of the NGF.

To validate the neuroprotective effect of our NGF-based nanoformulation, human retinal pigment epithelium ARPE-19 cells were incubated with hydrogen peroxide, to mimic an oxidative condition as occurs in retinal degeneration (Figure 4c). Cell viability was assessed by measuring the metabolic activity by performing an MTT assay after 24 h of co-incubation of cells with hydrogen peroxide and NGF-conjugated nanoparticles or free NGF. As expected, data showed a cytotoxic effect in the H_2_O_2_-treated group compared to the untreated control (****, *p* < 0.0001) as oxidative damage arose. While free neurotrophin was not able to counteract H_2_O_2_-induced cell mortality, cellular metabolic activity was preserved by nanoformulated NGF (ANP:PNA:NGF) compared to the H_2_O_2_-treated group (****, *p* < 0.0001), suggesting a pronounced protective effect only with the NGF nanoformulation.

### 3.4. Nanoformulated NGF Improves Visual Function in Zebrafish Larvae and Partially Protects Retinal Cells from Oxidative Stress-Triggered Apoptosis

**Figure 5:**
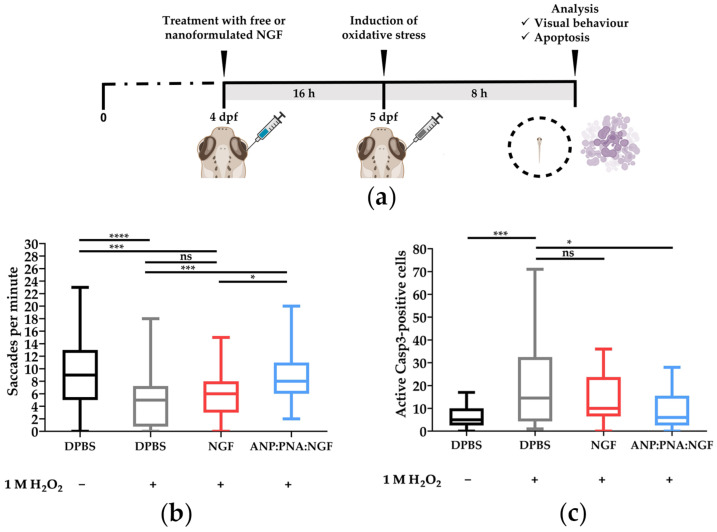
Nanoformulated NGF improves visual function impaired by oxidative stress in zebrafish larvae. (**a**) Experimental timeline relative to the intravitreal injection of free NGF or NGF bound to PNA-conjugated polyacrylamide nanoparticles (ANP:PNA:NGF) in zebrafish larvae at 4 days post-fertilisation (dpf), followed by H_2_O_2_ exposure to induce oxidative stress in the retina. As control, zebrafish larvae were IVT injected with DPBS. (**b**) Evaluation of visual function by OKR assay in zebrafish larvae exposed to the treatments shown in panel (**a**). *n* ≥ 39 larvae for each group, Kruskal–Wallis followed by Dunn’s multiple comparisons test. (**c**) Quantitative evaluation of apoptosis through the detection of active caspase 3 (Casp3)-positive cells by immunofluorescence in zebrafish larvae exposed to the treatments shown in panel (**a**). *n* ≥ 20 larvae for each group, one-way ANOVA using Dunnett’s multiple comparisons test. (**b**,**c**) ns, *p* > 0.05; *, *p* < 0.01; ***, *p* < 0.001 and ****, *p* < 0.0001.

To further confirm the neuroprotective activity of the ANP:PNA-conjugated NGF, in vivo experiments resembling a condition of retinal degeneration were performed in zebrafish larvae, by inducing a retinal oxidative damage previously developed by our research group [28]. Free NGF or nanoformulated neurotrophin (ANP:PNA:NGF) was IVT injected as a preventive treatment against an oxidative injury obtained through the intravitreal injection of hydrogen peroxide. To evaluate the effect of the nanoformulated neurotrophin in preventing vision impairment, visual function of injected larvae was evaluated by the optokinetic response (OKR) assay (Figure 5a).

Data showed a statistically significant reduction (****, *p* < 0.0001) in the ocular movements (saccades) recorded in the larvae injected with H_2_O_2_ (4.65 ± 0.51 saccades per minute) compared to the negative control group (9.42 ± 0.63 saccades per minute), thus confirming a visual impairment of larvae exposed to the oxidative stress. Injection of free neurotrophin was not able to prevent the visual dysfunction induced by hydrogen peroxide (5.81 ± 0.49 saccades per minute; ns, *p* > 0.05). Interestingly, the number of saccades recorded in ANP:PNA:NGF-injected larvae (8.59 ± 0.69 saccades per minute) was significantly higher compared to both the group treated with only hydrogen peroxide (***, *p* = 0.0001) and the one pretreated with free NGF (*, *p* = 0.0179), thus suggesting a neuroprotective effect of the NGF only when nanoformulated with the ANP:PNA nanocarrier. Additionally, no differences were observed between the larvae injected with ANP:PNA:NGF and the negative control group (ns, *p* > 0.05). In contrast, the number of saccades displayed by the larvae pretreated with free NGF was significantly lower than those of the negative control group (***, *p* = 0.0003) (Figure 5b).

The preventive treatment with neurotrophin showed promising results also against visual impairment induced by the exposure of zebrafish larvae to cigarette smoke extract (CSE). The number of saccades recorded in the negative control (28.44 ± 1.13 saccades per minute) was significantly higher (****, *p* < 0.0001) compared to the CSE-treated positive control group (13.68 ± 0.98 saccades per minute), in line with reported data [5]. Preventive injections of free NGF (21.96 ± 1.09 saccades per minute) or its nanoformulation ANP:PNA:NGF (20.19 ± 1.33 saccades per minute) significantly improved visual function impaired by exposure to the CSE (****, *p* < 0.0001 and ***, *p* = 0.0004, respectively) (Figure S3). This rescue was only partial since there was still a significant difference compared to the vehicle control group (**, *p* = 0.0017 and ****, *p* < 0.0001, respectively).

To study the mechanism underlying the recovery of compromised visual function induced by oxygen peroxide exposure, the neuroprotective effect of the NGF-conjugated nanoparticles was investigated at a functional level through the detection of active caspase 3 (aCasp3)-positive cells as a marker of apoptosis, as a consequence of an oxidative stress condition [44,45]. In agreement with the OKR data, the number of apoptotic cells was significantly higher (***, *p* = 0.0003) in the H_2_O_2_-treated group (20.8 ± 4.4 aCasp3-positive cells) compared to the negative control (6.2 ± 0.9 aCasp3-positive cells), hence corroborating cytotoxic damage induced by the injection of hydrogen peroxide. Interestingly, the reduction of apoptotic cells in the retina was only observed in the H_2_O_2_-injected larvae pretreated with ANP:PNA:NGF (9.7 ± 2.02 aCasp3-positive cells; *, *p* = 0.0113) while no neuroprotection was observed in the larvae pretreated with the free neurotrophin (13.8 ± 2.32 aCasp3-positive cells), since the number of apoptotic cells was not statistically different (ns, *p* > 0.05) to the group exposed to hydrogen peroxide (Figure 5c). These data are in line with the visual impairment revealed by the optokinetic response assay and show a promising effect of our neuroprotective NGF-based nanoformulation against the retinal degeneration induced by oxidative stress.

## 4. Discussion

This study aimed to develop a neuroprotective, polymeric-based drug delivery system as a preventive strategy against retinal degeneration or dysfunction. Due to the high loading efficiency, biocompatibility and biodegradability, we synthesised and optimised polyacrylamide-based nanoparticles for implementing an ocular drug delivery system for the posterior segment of the eye. To our knowledge, this is the first polymer-based nanoformulation implemented for delivering to the ocular posterior segment by both exploiting peanut agglutinin as a targeting molecule and the neurotrophin NGF as a neuroprotective compound.

The advancement of nanotechnology-based strategies in ophthalmology is one of the most popular approaches for achieving successful therapeutic options against vision loss, thanks to their significant abilities in improving drug delivery [20]. In particular, polymers are extensively employed as materials for drug delivery systems because of their noticeable biodegradability, stimuli-responsiveness, mucosal adhesive properties and biocompatibility [46,47]. Indeed, the number of polymer-based FDA-approved nanotechnological products for ocular treatment is increasing, but topical delivery still represents the preferred route of administration [47]. Unfortunately, the static and dynamic barriers that protect the eye pose many challenges, and only a small fraction of administered drugs (less than 5% of the instilled dose) reaches the retinal layers [48]. Consequently, topical instillation is not the optimal route for treating the posterior segment. Moreover, the majority of available ocular nanoformulations in the pharmaceutical market focus on the treatment of the anterior segment of the eye (dry eye disease, inflammation, acute keratitis, bacterial conjunctivitis and uveitis), thus not addressing retina degeneration in the posterior eye [21]. Since oxidative stress is the leading cause of cellular death and visual impairment, and it represents a common pathological mechanism in ocular degenerative diseases, great attention is addressed to developing neuroprotective systems for preventing retinal degeneration [1].

Here, we developed a multifunctional polymer-based ocular drug delivery system, and we assessed its neuroprotective effect in vivo, using a zebrafish model of retinal degeneration induced by oxidative stress, as previously developed by our group [28]. The synthesis of polyacrylamide nanoparticles embedding a fluorescent dye led to obtaining a polymeric nanosystem that was easily traceable in transparent zebrafish larvae, allowing in vivo imaging to study the localisation of the particles after intravitreal injections in larvae at 3 days post-fertilisation. Our results indicated that our polymeric nanoparticles spontaneously migrate through the retinal layers over time, as suggested by their presence mainly at the level of the ganglion cell layer after 4 h post-injection and their autonomous distribution towards photoreceptor and retinal pigment epithelium layers after 24 hpi. Importantly, no particles were found to diffuse in the contralateral eye. Unfortunately, in vivo imaging highlighted the presence of nanoparticles at the level of the pronephros, the ancestral kidney of vertebrate embryos, where they started accumulating from 4 hpi. Due to their instability in the eye, we initiated an optimisation of the nanocarrier by exploiting the easy functionalisation of the polyacrylamide nanoparticles, thanks to the presence of several functional groups on their surface [36,38]. Therefore, we selected peanut agglutinin (PNA) to take advantage of its specific and well-characterised high affinity for the galactose-galactosamine disaccharide residues of cone photoreceptors, as demonstrated in humans as well as in fish [42]. In fact, this is the first study where the PNA was nanoformulated for both stabilising a polymeric nanocarrier in the ocular environment and conferring an active targeting capacity, thus improving its applicability as an ocular drug delivery system. After the characterisation of the red fluorescent ANP:PNA, revealing both an increase in the hydrodynamic diameter compared to the naked formulation and good conjugation properties, in vivo studies showed a promising modification in the biodistribution of our nanocarrier. The presence of the fluorescent dye Alexa Fluor 594 contained in the polyacrylamide matrix and of an FITC fluorophore conjugated to the PNA easily allowed us to simultaneously follow the destiny of our nanocarrier and its cargo, respectively. After the intravitreal injection in 3 dpf larvae, the PNA-conjugated polymeric nanoparticles stably localised in the injected eye over time, without spreading in the contralateral eye or in any other larval tissues. On the contrary, naked ANPs were found in the pronephros at 24 hpi, thus suggesting their migration from the eye. The strong improvement in the localisation profile of the PNA-conjugated ANPs strongly suggests the ability of the lectin to prolong the residence time of the polymeric nanocarrier and its stability. Although the red signal from Alexa Fluor 594-positive nanoparticles and the green fluorescence associated with the FITC-conjugated PNA perfectly colocalise in the eye of larvae after 4 h post-injection, the two fluorescent signals showed an incomplete overlapping at 24 hpi. This can be explained by partial detachment of PNA from the ANPs occurring at 24 hpi, which can be ascribed to the fact that the functionalisation reaction involved noncovalent interactions and the ANP:PNA are exposed to physiological processes at the humour vitreous of the zebrafish eye.

Taking advantage of the evidence collected, we decided to perform a double conjugation onto the polyacrylamide nanoparticles’ surface using the NGF as a neuroprotective molecule against retinal degeneration. The role of this neurotrophin in the differentiation and maintenance of neurons is widely acknowledged. Indeed, several studies demonstrated that the NGF exerts prosurvival and regenerative effects in preclinical and clinical models of glaucoma [13,49,50], retinitis pigmentosa [51,52] and AMD [53] through the activation of different molecular pathways [14,15,16]. Although there is a robust body of evidence reporting the beneficial effect of the neurotrophin against retinal degeneration, NGF-based therapies are at the very early stages of market approval and clinical applicability due to several drawbacks that still require a solution, such as poor solubility, low delivery efficiency, short half-life and off-target effect [17]. To overwhelm these limitations, our group previously implemented an inorganic nanoformulation carrying the NGF [28]. Our previous results demonstrated increased stability and protection from degradation, resulting in increased prevention of retinal ganglion cell loss, upon conjugation of NGF with magnetic nanoparticles. Although these NGF-functionalised magnetic nanoparticles emerged as a promising ocular-tailored therapeutical nanotool, we decided to move towards an organic drug delivery system to overcome some disadvantages associated with the lack of evidence concerning biodegradability, toxicity and clearance mechanisms, which still pose some issues to the use of iron-based nanomaterials for the treatment of retinal disorders/degeneration.

Hence, our PNA-targeted polyacrylamide-based nanocarrier has been further functionalised with NGF via a noncovalent reaction, revealing a shift in the hydrodynamic diameter, an improved polydispersity index and a high conjugation efficiency. The bioactivity assessment of the nanoformulated NGF demonstrated its unaltered capacity, in triggering the differentiation of PC12 cells in a neuron-like phenotype compared to the free factor, thus suggesting that the conjugated NGF was biologically active. Further, to test the neuroprotective effect of our nanocarrier, we induced a condition of oxidative stress in human RPE cells by co-incubating hydrogen peroxide with free or ANP:PNA-conjugated NGF. In sharp contrast to evidence reported in the literature [54], we found that only the nanoformulated NGF, but not the free form, was able to exert a protective effect against the hydrogen peroxide-elicited oxidative damage, as indicated by the higher percentage of metabolically active cells in the ANP:PNA:NGF cotreated group.

To corroborate these encouraging results regarding the biological activity of the nanoformulation, we decided to validate the neuroprotective ability of NGF-conjugated ANPs in vivo using zebrafish larvae. Zebrafish represent a popular and well-established model for studying retinal degeneration or dysfunction due to the high genome homology and the same retinal stratification of different cell types shared with humans [23,24,25]. Moreover, since its development is extremely rapid, the visual function starts only 3 days post-fertilisation, thus allowing visual behavioural assessments and short-term studies in the early stages of development. Indeed, zebrafish constitute a valuable model organism for biomedical research and are increasingly being employed to conduct nanotoxicological studies and preclinical validation of innovative drug delivery nanoformulations [55,56]. Consequently, the ethical impact is significantly reduced as zebrafish are considered a non-sentient organism until 5 dpf.

The neuroprotective activity of our polymeric NGF-conjugated nanoformulation was tested using an ocular model of oxidative stress in zebrafish, previously developed by our group [28]. As expected, the visual behavioural assessment carried out through the OKR assay revealed impaired vision in larvae IVT injected with hydrogen peroxide compared to the saline-injected group. Most importantly, only the larvae pretreated with nanoformulated NGF showed a significantly higher number of saccades, compared to both the positive control and the free NGF-pretreated group. Conversely, the preventive intraocular injection of the free NGF was not able to improve the visual function impaired by the induction of oxidative injury. These results are in line with our previous findings that demonstrated an improved neuroprotection capability of the neurotrophin loaded in a drug delivery system, thus guiding us to speculate on increased stability and bioavailability of the nanoformulated NGF compared to the free form. In line with the data collected from the behavioural analysis, we also found a strong increase in the number of apoptotic cells in the retina of larvae IVT injected with hydrogen peroxide compared to the negative control group. These results are in line with evidence reported in the literature, according to which the apoptotic event and, in particular, the activation of specific caspase is strictly related to retinal degeneration as a consequent result of oxidative stress [44,45]. Most encouragingly, the preventive treatment with the nanoformulated NGF, but not the injection of the free NGF, resulted in a statistically significant reduction of active caspase 3-positive cells in the retina of injected larvae compared to the positive control, thus suggesting the ability of our nanoformulated NGF to prevent oxidative stress-triggered apoptosis.

Moreover, using a zebrafish model of CSE-induced visual impairment, we demonstrated that our NGF-based nanoformulation was able to improve the optokinetic response of zebrafish larvae compromised by acute systemic treatment with CSE. Thus, taking advantage of a zebrafish model of pharmacological modulation of visual behaviour, we were able to further assess the neuroprotective efficacy of our nanocarrier, paving the way for the development of neurotrophin-based therapeutical strategies against CSE-induced retinal degeneration. Although no difference emerged between the use of free NGF and its polymeric nanoformulation, this was not unexpected due to the acute experimental time frame. Notably, this is the first study demonstrating neurotrophin NGF rescue of visual function in zebrafish larvae negatively affected by systemic exposure to CSE. Additional studies will be performed to clarify the beneficial role of our polymeric NGF-based nanoformulation compared to the free neurotrophin in providing neuroprotection against a chronic exposure to CSE.

## 5. Conclusions

In this study, we developed a novel multifunctional polymeric nanoformulation, which represents a promising ocular drug delivery alternative for preventing retinal degeneration. Particularly, this is the first study demonstrating that the lectin PNA not only improves the targeting to the posterior segment of the eye but also prolongs the residence time of our NGF-conjugated nanocarriers, thus resulting in a neuroprotective response against oxidative stress, as observed both in vitro and in vivo in zebrafish. The intravitreal administration route of a nano-based system could overcome the requirement of multiple injections due to the unfavourable kinetics of the traditional form of drugs, thereby reducing side effects. However, further experiments are needed to investigate how long our nanocarrier can protect NGF and ensure its sustained neuroprotective release, as well as to perform a deeper toxicological assessment. Moreover, we demonstrated for the first time that nanoformulated NGF improves visual function of zebrafish larvae exposed to cigarette smoke extract, thus offering a potential treatment to be further validated and exploited against retinal dysfunction. Future perspective aims to extend the study to rodent models to acquire new evidence about our nanoformulation’s safety and efficacy profile.

## Figures and Tables

**Table 1:** Nanoparticle characterisation. Hydrodynamic diameter and polydispersity index of naked and functionalised polyacrylamide nanoparticles. Mean ± SEM, *n* = 3.

Nanoparticles	Functionalisation	Hydrodynamic Diameter	Polydispersity Index
ANPs	Alexa Fluor 594	13.89 ± 1.74 nm	0.44 ± 0.03
ANP:PNA	Alexa Fluor 594; PNA	19.97 ± 2.41 nm	0.47 ± 0.09
ANP:PNA:NGF	Alexa Fluor 594; PNA; NGF	22.81 ± 3.17 nm	0.18 ± 0.03

## Data Availability

The data presented in this study are available on request from the corresponding authors.

## Supplementary Materials

The following supporting information can be downloaded at https://www.mdpi.com/article/10.3390/pharmaceutics15041096/s1, Figure S1: the intravitreal injection of hydrogen peroxide induces apoptosis in the zebrafish retina, Figure S2: nanoparticles do not diffuse in the contralateral eye after intravitreal injections, Figure S3: intravitreal injection of ANP:PNA-conjugated NGF attenuates cigarette smoke extract (CSE)-induced visual impairment in zebrafish larvae.
