# Supplementary material for: Neuroprotective Nanoparticles Targeting the Retina: A Polymeric Platform for Ocular Drug Delivery Applications
Source: Pharmaceutics. 2023 Mar 29;15(4):1096. doi: 10.3390/pharmaceutics15041096 (PMC10144786; doi:10.3390/pharmaceutics15041096)
Supplement: Supplementary file 1 [file pharmaceutics-15-01096-s001.zip › Supplementary Figure Legends.pdf]

Supplementary Figure Legends

Figure S1. The intravitreal injection of hydrogen peroxide induces apoptosis in the zebrafish retina. Representative image of the retina of a larva at 5 days post-fertilization where apoptotic cells were detected by immunostaining using an anti-active caspase 3 antibody. White arrows indicate apoptotic cells (cyan). Cell nuclei stained with Hoechst 33342 (blue). Scale bar: 50 μm.

Figure S2. Nanoparticles do not diffuse in the contralateral eye after intravitreal injections. Representative images of a retinal cross-section of a zebrafish larva intravitreally injected with Alexa Fluor 488-positive polyacrylamide nanoparticles, analysed at 24 hours post injection (hpi). Cell nuclei stained with Hoechst 33342 (blue); retinal distribution of nanoparticles revealed by the fluorescent dye Alexa Fluor 488 (green). Scale bar: 200 μm.

Figure S3. Intravitreal injection of ANP:PNA-conjugated NGF attenuates cigarette smoke extract (CSE)-induced visual impairment in zebrafish larvae. Evaluation of visual function by OKR assay in zebrafish larvae pre-treated with free or nanoformulated NGF and exposed to cigarette smoke extract. Negative control group was intravitreally injected with DPBS and treated with 0.05% DMSO solution. $n \geq 25$ larvae for each group, one-way ANOVA by Tukey's multiple comparisons test. **, $p<0.01$; ***, $p<0.001$; ****, $p<0.0001$.
