# Supplementary figures and images for: Neuroprotective Nanoparticles Targeting the Retina: A Polymeric Platform for Ocular Drug Delivery Applications
Source: Pharmaceutics. 2023 Mar 29;15(4):1096. doi: 10.3390/pharmaceutics15041096 (PMC10144786; doi:10.3390/pharmaceutics15041096)

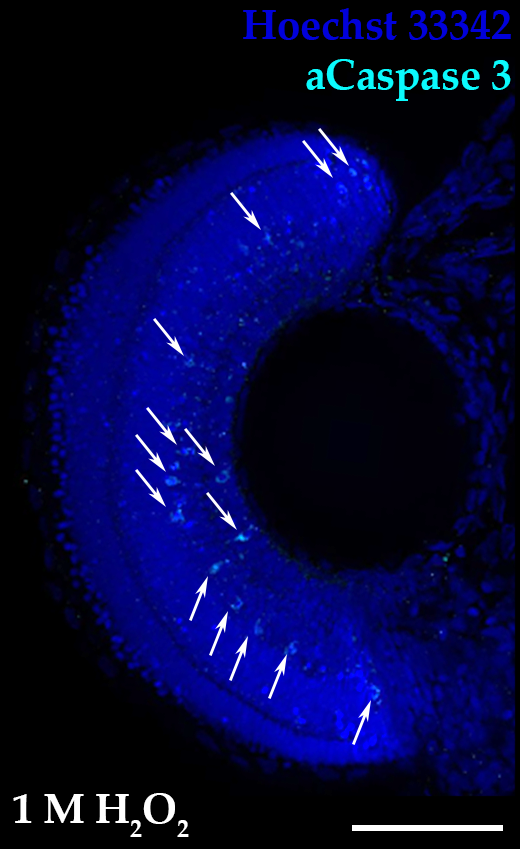

Supplement: Supplementary file 1 [file pharmaceutics-15-01096-s001.zip › Figure S1.tif]

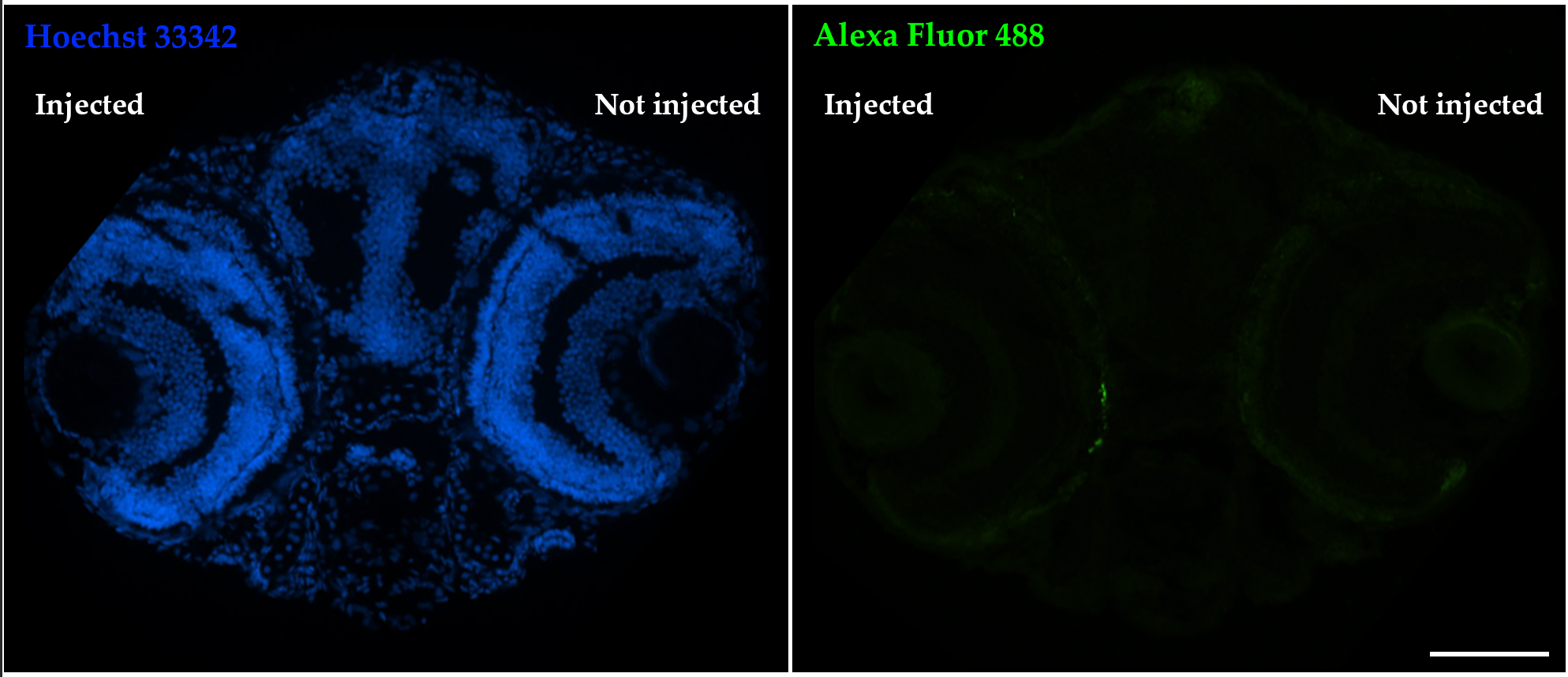

Supplement: Supplementary file 1 [file pharmaceutics-15-01096-s001.zip › Figure S2.tif]

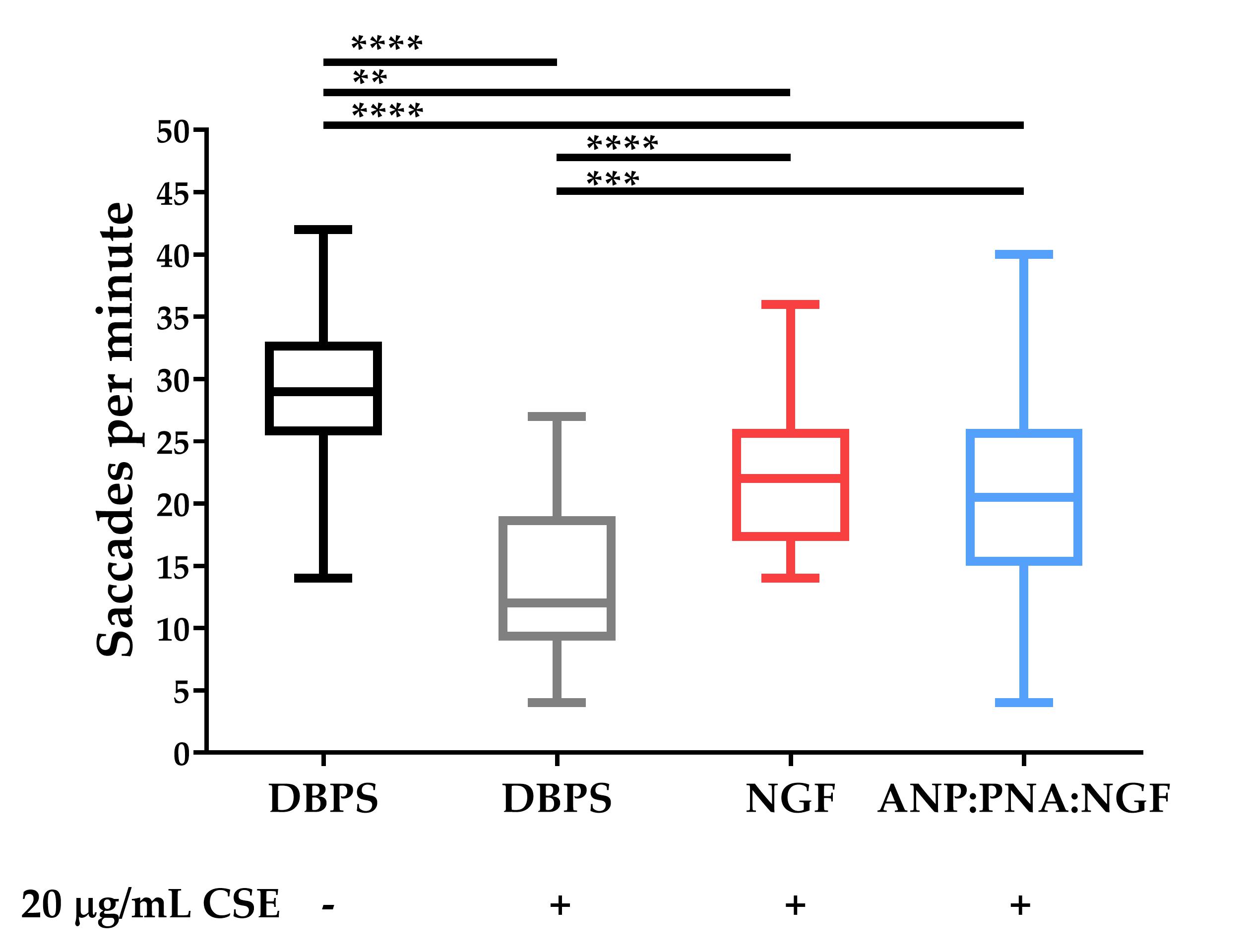

Supplement: Supplementary file 1 [file pharmaceutics-15-01096-s001.zip › Figure S3.jpg]
